# Determinants of problem sports betting among college students: moderating roles of betting frequency and impulsive betting tendency

**DOI:** 10.1186/s40359-023-01387-w

**Published:** 2023-10-23

**Authors:** Yawen Shen

**Affiliations:** Department of Physical Education, Zhongyuan University of Technology, No. 41 Zhongyuan Rd, 450007 Zhengzhou, Henan Province China

**Keywords:** Sports betting, Problem gambling, Impulsive betting, Theory of planned behavior

## Abstract

**Purpose:**

Given the risk and increased incidence of problem betting for young adults, the purpose of the current study was to understand what influences college students’ problem sports betting behavior using the theory of planned behavior (TPB).

**Methods:**

An institutional-based cross-sectional study was conducted. Data were collected from 311 college students in the U.S. using a survey questionnaire and primarily analyzed using the partial least squares structural equation modeling (PLS-SEM) technique to investigate the relationships between the study variables. In addition, multi-group SEM analyses were conducted to investigate the moderating roles of betting frequency and impulsive betting tendencies regarding sports betting.

**Results:**

The results suggested that college students’ sports betting intentions (SBI) were associated with attitude towards sports betting, motivation to comply with others, and subjective norm, in this order, but not with perceived behavioral control (PBC). Problem sports betting (PSB) was significantly positively related to their SBI and negatively correlated with PBC. In addition, multigroup analyses found the moderating roles of betting frequency and impulsive betting tendency, especially in the relationship between SBI and PSB. The SBI-PSB relationship was stronger with the infrequent/low-betting group and low-impulse betting group, compared to the frequent/high-betting and high-impulse betting group.

**Conclusion:**

Overall, the results highlighted the importance of peer influence and attitude formation concerning sports betting. Recognizing what influences PSB and the roles of habitual and impulse sports betting in this population are recommended in developing proper public health programs to mitigate PSB issues.

**Supplementary Information:**

The online version contains supplementary material available at 10.1186/s40359-023-01387-w.

## Introduction

Given the lasting influence of problem gambling or betting (hereafter, problem betting) on younger populations and the increase in the size of the sports gambling industry [1], it is critical to understand what influences the college-aged population’s sports betting-related behavior [1–4]. In the U.S., as the NBA commissioner mentioned, it is inevitable to have ‘expanded legalized sports betting’ [5]. Also, betting firms’ investment in sports in terms of corporate sponsorship has also increased over the last few decades [6, 7]. In countries like the U.K., where the sports betting culture has been established for a long while, there are more people who have problems with their sports betting and other related issues. For example, the number of problem bettors has increased, especially since many younger adults have displayed signs of a betting problem [8–10].

The theory of planned behavior (TPB) has been applied to understand behavioral intention and/or overt behavior, including betting-related intention and behavior [11, 12]. While there are a sizable number of studies on college students’ gambling using TPB [13, 14], not many researchers have investigated the issue of sports-related problem betting [6]. For one of the TPB variables, namely subjective norms, the current study used a two-component construct/measure of subjective norms, including normative beliefs and motivation to comply with others because college students are still vulnerable to peer influence, given their life settings and age. Also, the moderating roles that individual impulsive betting tendencies and previous sports betting experiences (i.e., betting frequency) play in problem sports betting were explored.

Thus, the current study evaluated what predicts college students’ sports betting behavior, including sports betting intention and problem sports betting behavior, using the theory of planned behavior [11, 12], i.e., subjective norm, attitudes towards sports betting, perceived behavioral control, and motivation to comply with others. The current study also incorporated non-volitional factors (e.g., impulsive sports betting tendency), which are often overlooked in TPB-based studies, to better understand sports betting-related behavior among college students.

### Sports betting and problem betting behavior

Since a pivotal Supreme Court ruling in 2018, each state has the authority to legalize sports betting in the United States [15]. Consequently, about two-thirds of U.S. states have approved some form of legalized sports betting, and about 80% of them have either legalized or at least proposed bills to legalize sports betting. For example, Ohio’s bill was passed in 2021 and is expected to be effective in 2023 [16, 17]. Not surprisingly, the number and frequency of sports betting in the U.S. have increased over the years. In December 2021, 24% of Americans participated in sports betting, and 12% of them waged in sports at least weekly [16]. Sports betting revenue has grown significantly since legalization, from $0.43 billion in 2018 to $4.33 billion in 2021. Since its inception in 2018, more than $125 billion was waged as of May 2022 [16]. According to the National Council on Problem Gambling (NCPG), those who bet on sports are twice more likely to have betting problems than gamblers who do not bet on sports [18].

Also, online sports bettors are more likely to have problem betting issues than offline/in-person sports bettors. In fact, among online sports bettors, about 29% are assumed to have gambling problems or gambling disorders [18]. Given the legalization trend and projected revenue growth of sports betting in upcoming years, it is expected to have a greater level of problem betting issues in the U.S., especially with younger (up to age 35) male populations [9, 18, 19]. Therefore, it is imperative to understand what influences problem betting behavior among (potential) sports bettors in order to promote healthier sports betting behavior.

### Theory of planned behavior and sports gambling behavior

The TPB is one of the most effective frameworks for understanding what influences individual intentions to engage in a particular behavior [12]. The TPB posits that individual attitude (i.e., how people evaluate a focal behavior), subjective norm (i.e., how people perceive their significant others’ evaluation of a focal behavior), and perceived behavioral control [PBC] (i.e., how people appraise their own control over a focal behavior or how they perceive their self-efficacy toward a particular behavior) determine their intention to engage in a particular behavior [11, 12]. Thus, an individual is likely to have a higher intention to perform a particular behavior if he or she has a positive attitude toward a particular behavior, a positive social norm concerning this behavior, and a good sense of strong behavioral control over performing a particular behavior [12]. Likewise, a (potential) sports bettor is likely to engage in sports betting activities if the individual has a positive attitude toward sports betting, is surrounded by significant others who are supportive of sports betting, and has control over his or her sports betting activities.

The TPB has been utilized to understand gambling and betting-related behavior among young adults [14, 20–23]. For example, Wu and Tang, with a sample of college students in Hong Kong and Macao, found that the intention to gamble was predicted most by attitudes toward gambling, followed by subjective norms and PBC, while problem gambling was significantly correlated with intention and PBC [14]. Using an Australian online research panel, Flack and Morris found that subjective norms (i.e., normative beliefs) were the strongest determinant of intention to gamble, compared to other TPB variables, while intentions to gamble were a significant predictor of gambling frequency [20]. In a Canadian context, St-Pierre et al. found that gambling intention was significantly correlated with attitudes toward gambling and PBC over gambling refusal but not with subjective norms of family and peers on gambling [21]. In addition, bettors’ gambling frequency was significantly associated with the intention to gamble, followed by attitudes toward gambling, while their perceived gambling problems were better predicted by PBC, followed by the intention to gamble. Overall, St-Pierre et al.’s study suggested the efficacy of TPB in predicting gambling behavior, including problem gambling [21].

Similarly, Martin et al. found that TPB determinants were effective predictors of both past gambling behavior and gambling intention [22]. Specifically, the intention to gamble was most closely associated with the friend/family subjective norms, followed by attitudes and PBC. In a similar context of casino gambling, Lee found that college students’ favorable attitudes toward and perceived support for casino gambling, along with gambling media exposure and prior gambling experience, were significant determinants of casino gambling intentions [23]. However, the perceived behavioral control about casino gambling was not a significant proximal predictor in Lee’s study with undergraduate students in the U.S.

More specifically, regarding sports betting, Wang et al. claimed that the TPB framework could be utilized to predict sports betting behavior [24]. They found that attitude and subjective norms were critical antecedents to college students’ intention to bet on sports, while their intention and PBC were proximal determinants of their sports betting behavior. In a Finnish context, Kekki found that young adults’ intention to play sports betting was associated with PBC, subjective norms, and attitude towards playing sports betting, in this order, along with reasons and motivation to play [25]. In an Australian context, Hing et al. found that the intention to bet on sports in the next six months was significantly influenced by positive attitudes towards sports betting participation and positive subjective norms about sports betting, especially when they had more frequent exposure to the promotions [6].

### Study hypotheses

As discussed, the efficacy of the TPB in explaining gambling or betting behavior has been well supported by the literature [2, 14]. However, relatively little attention has been paid to understanding what influences college students’ problem (sports) betting behavior. Therefore, the current study utilized the TPB framework to investigate the relative importance of TPB determinants in explaining college students’ problem sports betting behavior (PSB).

**Fig. 1:**
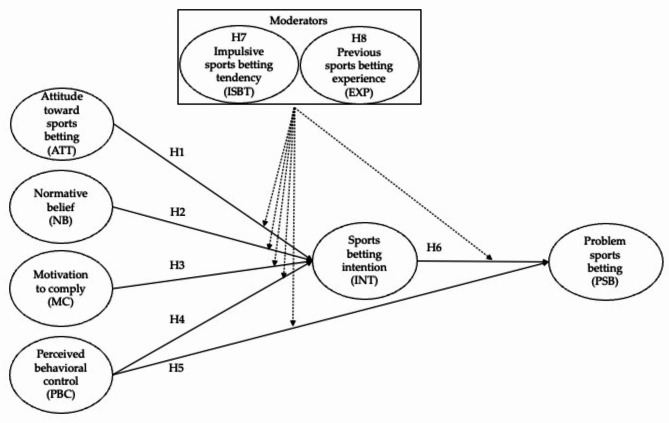
Theoretical model and hypotheses in this study

According to the TPB theory and literature [11, 12], it is posited that college students’ intention to participate in sports betting would be positively associated with favorable attitudes towards sports betting and favorable perceptions of significant others (i.e., subjective norms) while negatively associated with PBC concerning sports betting. Regarding social norms concerning sports betting, this study utilized two components of the subjective norm: (1) expectations of significant others and (2) motivation to comply with others, given the peer influence on this population in this particular context [22, 26]. In addition, the TPB framework posited that college students’ sports betting intention would mediate the relationship between TPB distal determinants (i.e., attitudes, normative beliefs, motivation to comply, and PBC) and PSB, while one of the distal determinants (namely, PBC) would be directly correlated with PSB [12]. Therefore, the following hypotheses were suggested and subsequently tested in this study (refer to Fig. 1):

#### Hypothesis 1

(H1): College students’ intention to bet on sports is associated with their favorable attitude to sports betting.

#### Hypothesis 2

(H2): College students’ expectations of significant others influence sports betting intention.

#### Hypothesis 3

(H3): College students’ motivation to comply with others influences sports betting intention.

#### Hypothesis 4

(H4): College students’ perceived behavioral control influences sports betting intention.

#### Hypothesis 5

(H5): College students’ perceived behavioral control influences problem betting behavior.

#### Hypothesis 6

(H6): College students’ sports gambling intention influences problem betting behavior.

A review of the literature also found some inconsistencies in the relative importance of TPB determinants in explaining betting-related behavior. For example, Wang et al. would argue that attitude is the most critical determinant, while Kekki could claim PBC is more important in predicting college students’ intention of sports betting [25]. It can be argued that there exist some impactful moderators, such as impulsive betting tendencies and previous betting experience (i.e., betting frequency), that influence the relative importance of TPB determinants on sports betting-related behavior [27, 28].

The TPB is based on the assumption that human beings are usually rational and, thus, make good use of the information available to them [12]. However, it is possible that people would have an impulsive betting tendency, the degree to which an individual is likely to make unintended and unreflective bets on sports. Similar to the concept of impulse buying [29], impulse betting is likely to occur when bettors have “a predisposition toward rapid, unplanned reactions to internal or external stimuli without regard to the negative consequences of these reactions” [30; p. 1784]. Thus, the relative strength of the structural paths between TPB variables would differ depending on this individual tendency [28]. For example, the relationship between the intention and PSB would be stronger with the low impulsive tendency group, given that impulsive betting tendency involves unreflective betting. Also, it is possible that attitude would be the most important distal determinant of the low impulsive tendency group, while motivation to comply could be the most critical antecedent for the high impulsive tendency group. As such, it seemed that impulsive sports betting tendency (ISBT) is a meaningful moderator in understanding the TPB dynamics in the context of sports betting.

It is logical to assume that previous sports gambling experiences affect bettors’ motivations and expectations concerning sports betting [31]. A first-time or inexperienced bettor may develop an intention or make a decision to bet based on sports betting commercials and ads or peer word of mouth to a great extent, whereas a repeat or experienced bettor may be more influenced by his/her previous experiences with sports betting. Thus, it can be expected that less-involved or potential bettors are more influenced by social norms, while more-involved bettors are more influenced by their attitudes (e.g., perceived utilities) or betting frequency (i.e., habitual conduct). Therefore, the following hypotheses were formulated (refer to Fig. 1).

#### Hypothesis 7

(H7): College students’ impulsive sports betting tendency moderates the influences of TPB distal determinants on intention and problem sports betting behavior.

#### Hypothesis 8

(H8): College students’ previous sports betting experience moderates the influences of TPB distal determinants on intention and problem sports betting behavior.

## Methods

### Participants and data collection

Data collection was conducted using a convenience sampling method. College students were invited to complete an online questionnaire. Participants read an informed consent before answering the questions and completed the survey without a monetary reward. After removing four incomplete respondents, a total of 311 college students participated in this study, with 68.2% males (*n* = 212) and 31.8% females (*n* = 99). The average age of participants was 20.87 (*SD* = 2.31). About 75% of the respondents had previous experiences with sports betting, and thus, the vast majority of them were familiar with sports betting.

### Survey instrument

The survey questionnaire was developed based on the review of relevant literature, especially from gambling and betting-related studies that utilized the TPB framework [12, 24, 32, 33], and comprised seven measures, including attitude (ATT; five items), normative beliefs (NB; three items), motivation to comply (MC; two items), perceived behavioral control (PBC; two items), sports betting intention (INT; two items), problem sports betting behavior (PSB; four items), and impulsive sports betting tendency (ISBT; three items). The scales used in this study have demonstrated good validity and reliability in previous studies and are measured using a 7-point Likert scale, ranging from strongly disagree (1) to strongly agree (7). Examples of the survey items include “Sports betting is a good leisure activity” (ATT), “People important to me would approve of me betting on sports” (NB), “If people important to me bet on sports, I would also participate in sports betting” (MC), “I feel that I have complete control over betting on sports” (PBC), “I intend to continue betting on sports in the near future” (INT), “Sometimes I try to keep the amount I bet on sports secret from family or friends”(PSB), and “I often bet on sports even if I had not intended to do” (ISBT). The survey questionnaire also included respondents’ demographic information, such as age, gender, and betting frequency.

### Data analysis

Data were primarily analyzed using partial least squares structural equation modeling (PLS-SEM), with SmartPLS 3.3.2, given the main objective of the current study was testing a theoretical framework from a prediction perspective [34, 35]. This particular analytic method was used because the method does not require normally distributed data by default and it can maximize the explained variance in the outcome variables [34, 35]. Based on Hair et al.’s guidelines [34, 35]. Firstly, the measurement model was evaluated in terms of scale reliability and validity. Secondly, the structural model was assessed to test the hypothesized relationships in the research model. Lastly, a multi-group SEM was employed to test the moderating role of ISBT (high and low ISBT groups) and previous sports gambling experience (EXP) on the hypothesized relationships in this study. If the main objective of moderator analyses is to explore the moderation effects on the entire structural model, using PLS-MGA is preferred, compared to testing the moderation effects using the interaction term [36]. Consequently, per Hair et al’s recommendations, K-means cluster analysis was used to define meaningful subgroups of the respondents based on their betting/gambling experience (i.e., high and low EXP groups) because cluster analysis can increase the heterogeneity between groups while maximizing the homogeneity of groups within clusters [34]. In addition, the dividing point (i.e., percentiles) was used to dichotomize the ISBT variable, a continuous moderator [34].

The current study used the PLS algorithm, followed by the PLS bootstrapping algorithm (basic-corrected and accelerated bootstrap) with 2,000 subsamples to assess factor loadings, path coefficients, and significant levels [35, 37]. Instead of specifying model fit indices when obtaining structural model solutions, PLS-SEM mainly depends on a different set of indices, such as collinearity (VIF), construct reliability (e.g., composite reliability), construct validity (e.g., average variance explained and Heterotrait-Monotrait-Ratio), and prediction indices (e.g., the coefficient of determination) [35, 37].

## Results

### Descriptive statistics and correlations

Table 1 represents the means, standard deviations, and bivariate correlations of the study variables in this study. College students’ intention toward sports betting was significantly correlated with attitude (r = .63, p < .001), MC (r = .60, p < .001), NB (r = .54, p < .001), and PBC (r = .15, p < .01). Similarly, PSB was positively associated with intention (r = .60), attitude (r = .42), MC (r = .40), and NB (r = .31), all the 0.001 significant level. However, PSB was not significantly correlated with PBC.

**Table 1:** Descriptive statistics and correlations

Variables	ATT	NB	MC	PBC	INT	PSB
ATT						
NB	0.54***					
MC	0.57***	0.47***				
PBC	0.27***	0.25***	0.09			
INT	0.63***	0.54***	0.60***	0.15**		
PSB	0.42***	0.31***	0.40***	− 0.01	0.60***	
*M*	3.92	3.99	4.35	6.01	3.31	2.16
*SD*	1.43	1.43	1.63	1.25	1.86	1.31

***p < .001, **p < .01, *p < .05

*Note*: ATT = Attitude; NB = Normative belief; MC = Motivation to comply; PBC = perceived behavioral control; INT = Intention; PSB = Problem sports betting behavior

### Measurement model

Firstly, the measurement model was assessed in terms of internal consistency reliability (i.e., Cronbach’s alpha coefficients and composite reliability [CR]), convergent validity (i.e., factor loading, and average variance extracted [AVE]), and discriminant validity (i.e., Heterotrait-Monotrait-Ratio [HTMT]) [38]. Cronbach’s alpha values ranged from 0.65 to 0.89, all above the 0.65 threshold for survey studies [39, 40]. CR values ranged from 0.80 to 0.95, scoring well above the 0.70 threshold, indicating adequate internal consistency reliability [34, 41, 42]. Factor loadings ranged from 0.69 to 0.95, and AVE values ranged from 0.59 to 0.90, indicating adequate convergent validity [34, 42]. Lastly, all HTMT values were lower than the threshold value of 0.85, indicating adequate discriminant validity of the constructs included in this study [42].

### Structural model

Before testing the study hypotheses, the variance inflation factor (VIF) values were examined to check the multicollinearity of the structural model. The VIF values ranged from 1.01 to 2.75, below the suggested threshold value of 3.30 [35, 43], indicating an absence of collinearity and common method bias. The structural model assessment suggested that the model had a substantial explanatory capacity in explaining outcome variables, as it explained 60.0% of the variance in sports gambling intention and 40.9% of the variance in PSB.

As reported in Table 2, in terms of distal TPB determinants, the attitude had a positive influence on sports betting intention, β = 0.36, t = 7.15, p < .001, thus supporting H1. Normative belief and motivation to comply also had positive influences on sports betting intentions, β = 0.19, t = 4.19, p < .001 and β = 0.36, t = 7.23, p < .001, respectively, thus supporting H2 and H3. PBC had no significant direct on sports betting intention but was negatively associated with problem sports betting, β = − 0.17, t = 3.37, p < .001, thus rejecting H4 and accepting H5. Lastly, sports betting intention had a positive influence on problem sports betting behavior, β = 0.63, t = 17.80, p < .001, thus supporting H6.

**Table 2:** Direct and Specific indirect effects

Category	Path	*β*	*SD*	*t*-value
Direct paths	ATT → INT	0.36	0.05	7.15***
	NB → INT	0.29	0.04	17.80***
	MC → INT	0.36	0.05	7.23***
	PBC → INT	− 0.001	0.04	0.12
	PBC → PSB	− 0.17	0.05	3.37***
	INT → PSB	0.19	0.05	4.19***
Specific indirect paths	ATT → INT → PSB	0.23	0.04	6.48***
	NF → INT → PSB	0.12	0.03	3.95***
	MC → INT → PSB	0.23	0.03	6.94***
	PBC → INT → PSB	− 0.003	0.02	0.12

*Note*: ***p < .001, **p < .01, *p < .05

In addition, mediation analysis was also performed to estimate the indirect effects of distal TPB determinants on problem betting behavior. As reported in Table 2, the significant indirect effects of TPB determinants on problem betting indicated the existence of mediation. The results showed that three out of four specific indirect paths were significant (refer to Table 2).

### Multi-group analysis (PLS-MGA)

Before conducting multi-group analyses, independent t-tests were employed to investigate differences in the study variables (1) between low-impulsive (n = 199) and high-impulsive (n = 112) sports betting groups and (2) between less-experienced (n = 154) and more-experienced (n = 157) sports betting groups. Respondents were grouped based on their individual impulsive tendency scores (four items) and previous general and sports betting experiences (four items) using k-means cluster analyses. The results are reported in Table 3.

**Table 3:** Summaries of independent *t*-tests

	Mean (*SD*) per ISBT group				Mean (*SD*) per Experience group		
Constructs	High ISBT	Low ISBT	*t-*value		More experienced	Less experienced	*t*-value
Attitude	4.72 (4.42)	3.55 (1.28)	8.02***		4.65 (1.10)	3.27 (1.24)	10.40***
NB	4.54 (1.09)	3.71 (1.43)	5.35***		4.58 (1.11)	3.43 (1.38)	8.10***
MC	5.17 (1.15)	3.88 (1.63)	7.37***		5.16 (1.22)	3.51 (1.51)	10.55***
PBC	6.18 (1.02)	6.05 (1.01)	-1.10		6.28 (0.90)	5.98 (1.10)	2.60*
Intention	3.67 (1.40)	1.38 (1.59)	11.68***		4.57 (1.46)	2.16 (1.28)	15.43***
PSB	4.73 (0.96)	1.38 (0.48)	27.95***		2.78 (1.28)	1.61 (1.03)	10.42***

*Note*: ***p < .001, **p < .01, *p < .05

The high-ISBT group had significantly higher levels of favorable attitudes toward sports betting (M = 4.72; t = 8.02, p < .001), normative belief (M = 4.54; t = 5.35, p < .001), motivation to comply (M = 5.17; t = 7.37, p < .001), sports betting intention (M = 3.67; t = 11.68, p < .001), and PSB (M = 4.73; t = 27.95, p < .001) when compared to the low ISBT group. However, both low and high ISBT groups had a fairly high level of PBC (M = 6.05 and 6.18, respectively), and thus there was no statistical group difference. Regarding the group differences based on sports betting experiences, more experienced bettors had significantly higher levels of attitude (M = 4.65; t = 10.40, p < .001), normative belief (M = 4.58; t = 8.10, p < .001), motivation to comply (M = 5.16; t = 10.55, p < .001), PBC (M = 6.28; t = 2.60, p = .01), betting intention (M = 4.57; t = 15.43, p < .001), and PSB (M = 2.78; t = 10.42, p < .001). Please refer to Table 3 for more information.

Overall, the results indicated that college students with higher impulsive betting tendencies and relatively more sports betting experiences have a higher level of favorable attitudes towards sports betting, normative beliefs, and motivation to comply with others concerning sports betting, intentions to bet, and potential betting-related problems. One thing to be noted is that relative to other variables, the level of PBC was comparatively similar regardless of group memberships.

To investigate the moderating effects of impulsive sports betting tendency in the research model, the multi-group analysis approach was employed based on their individual impulsive tendency scores (i.e., high and low ISBT groups) to assess whether path coefficients vary between the two groups (see Table 4). Statistically, a significant difference was identified in the path between intentions and problem betting behavior (p = .031). More specifically, the intention had a stronger influence on problem betting behavior for the low-impulsive betting group (β = 0.60, t = 11.55, p < .001) in comparison to the high-impulsive betting group (β = 0.34, t = 2.11, p = .035). In terms of the relative importance of the TPB distal determinants in predicting their intentions, motivation to comply (β = 0.40, t = 6.25, p < .001) was the most critical determinant, followed by the attitude (β = 0.26, t = 4.40, p < .001) and normative beliefs (β = 0.20, t = 2.26, p < .001) for the low-tendency group, while their attitude (β = 0.34, t = 4.07, p < .001) was most important for the high-tendency group, followed by motivation to comply (β = 0.28, t = 3.35, p < .001) and normative beliefs (β = 0.19, t = 2.26, p = .024).

**Table 4:** Summaries of path coefficients by group

		High ISBT	Low ISBT	Path Diff.		More experienced	Less experienced	Path Diff.
Paths		*β* (*S.D*.)	*β* (*S.D*.)	*p*-value		*β* (*S.D*.)	*β* (*S.D*.)	*p*-value
ATT → INT		0.34*** (0.08)	0.26*** (0.06)	0.408		0.37*** (0.07)	0.23*** (0.07)	0.152
NB → INT		0.19* (0.08)	0.20** (0.06)	0.896		0.07 (0.07)	0.33*** (0.07)	**0.004**
MC → INT		0.28*** (0.08)	0.40*** (0.06)	0.281		0.35*** (0.08)	0.27*** (0.08)	0.473
PBC → INT		0.00 (0.07)	0.04 (0.07)	0.686		− 0.08 (0.12)	− 0.12 (0.09)	0.767
PBC → PSB		− 0.25 (0.22)	− 0.07 (0.10)	0.437		− 0.17 (0.12)	− 0.20* (0.09)	0.863
INT → PSB		0.34* (0.16)	0.60*** (0.05)	**0.031**		0.38*** (0.07)	0.61*** (0.07)	**0.029**

*Note*: ***p < .001, **p < .01, *p < .05

To examine the moderating effects of previous (sports) betting experiences (i.e., less-experienced and more-experienced groups), another multi-group analysis was conducted (refer to Table 4, right-hand columns). The results from PLS-MGA found statistical differences between the two groups in the paths between normative beliefs and intention (p = .004) and intention and problem betting (p = .029). Normative beliefs had a stronger and more significant influence on the intentions of the less-experienced group (β = 0.33, t = 5.03, p < .001), while it did not significantly influence the intentions of the more-experienced group (β = 0.07, t = 1.13, p = .258). The intentions had a stronger influence on problem betting behavior for the less-experienced group (β = 0.61, t = 8.36, p < .001) in comparison to the more-experienced group (β = 0.38, t = 4.79, p < .001).

## Discussion

Sport is a critical element in our culture, and in some countries such as the U.K., sports betting is part of daily life and a form of entertainment and lifestyle. However, sports bettors, especially young adult males, are subject to a greater risk of problem betting [44, 45]. Thus, understanding what influences problem sports betting among young adults is necessary to develop a healthier sports betting culture.

**Fig. 2:**
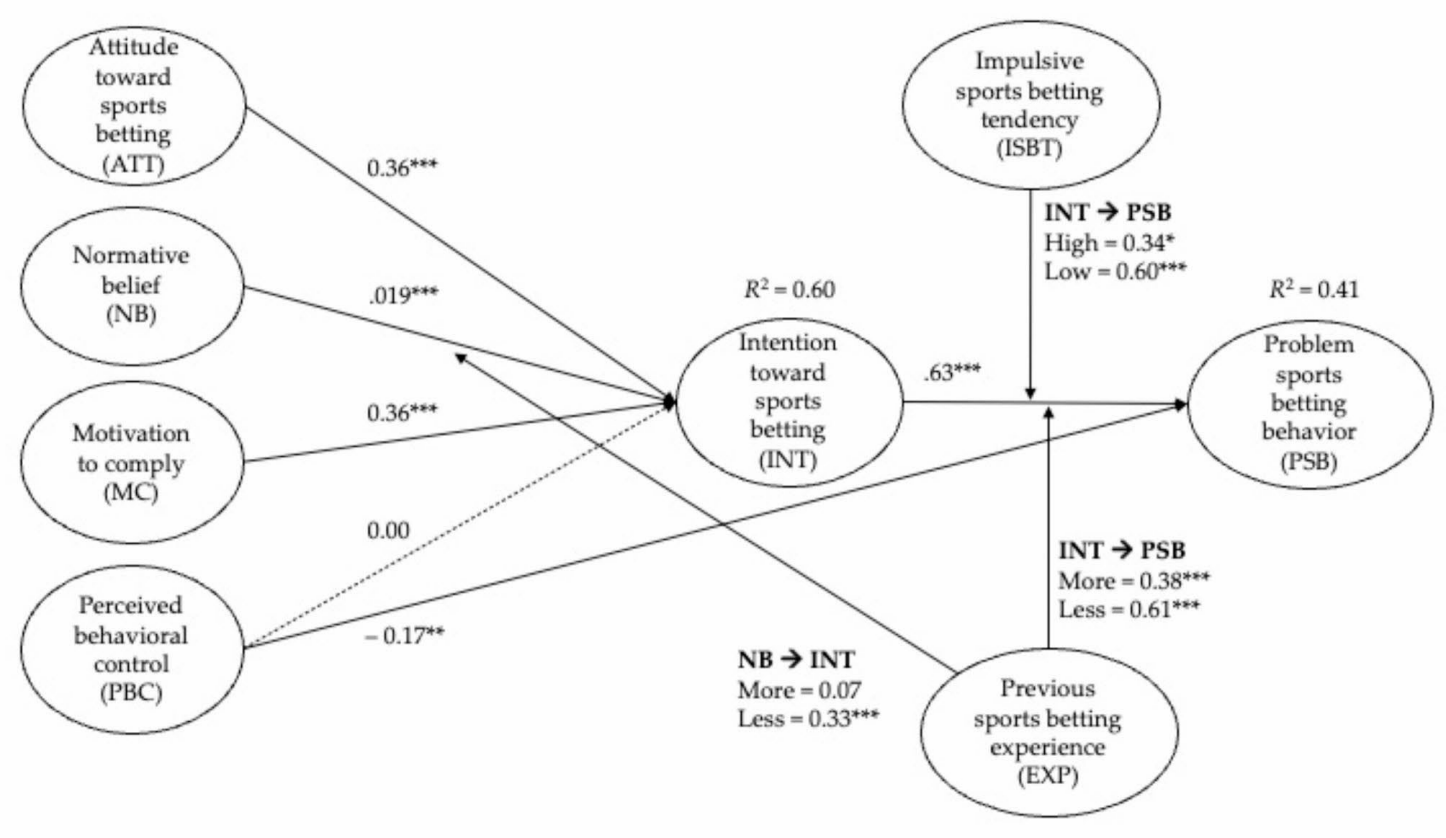
Final model with path coefficients *Note*: ***p < .001, **p < .01, *p < .05

The current study utilized the TPB framework to investigate the antecedents, mediators, and moderators for problem sports betting behavior among college students. The findings suggested that the distal TPB variables significantly contributed to explaining the proximal TPB variable (intentions) and college students’ intention toward sports betting, and PBC were effective determinants of their (potential) problem sports betting behavior. In addition, the current study suggests that impulsive betting tendencies and previous experiences of sports betting could be meaningful moderators in understanding the degree to which TPB determinants predict sports betting behavior. The findings provided support for the utility of the TPB and extended variables in the context of sports betting among college students. See Fig. 2 for the summarized results of the current study.

The intention to conduct sports betting was most closely related to college students’ attitudes toward sports betting, motivation to comply with others, and normative beliefs. The intention toward sports betting increases as the strength of their positive attitude and normative beliefs toward betting and motivation to comply with others increases. However, unlike our expectations, PBC was not directly related to sports betting intentions. While not all individuals with a high level of sports betting intentions would experience betting/gambling issues, it is likely that college students with a higher level of betting intentions and, thus, frequent actual betting behavior are more likely to experience betting-related problems. Therefore, understanding the determinants of sports betting intention and problem betting would be one of the critical tasks in developing a culture of and strategy for responsible betting [14, 22, 23, 45].

Consistent with the literature [8, 45, 46], the results suggest that attitude toward sports betting is a key determinant of betting intentions. In general, people hold negative attitudes toward betting, given the potentially harmful consequences [44]. However, due to the positive image and embeddedness of sports in our society, people tend to form a more favorable attitude toward sports betting [46]. Younger adults tend to have a relatively more positive attitude toward sports betting (e.g., sports betting livens up life) and a tendency to overestimate their chances of winning (i.e., overly positive cognitive attitude) because they are familiar with and identified with professional sports in comparison to other age groups. For example, Seal et al. claimed that sports fans with a favorable and permissive attitude toward sports, i.e., perceiving sports betting as harmless, common, and a part of sports, are more likely to bet on sports more frequently [45]. Consequently, health promotion and regulatory agencies should develop better educational campaigns to improve knowledge about the harmful consequences of the problem and pathological sports betting. Also, they should revisit how the media deals with information about sports betting, given the impact of the media’s influence on shaping the perceptions concerning sports betting [44, 46].

College students’ motivation to comply with others and their normative belief against sports betting were significant determinants of their sports betting behavior and potential betting problems. Relative to older counterparts, college students are likely to be more influenced by their peer groups. Gordon et al. used the concept of lifestyle consumption community (LCC) in explaining sports betting behavior among young adult non-pathological gamblers [47]. As young male adults are likely to have stronger and more diverse LCCs given their active lifestyle, they are more likely to be greatly influenced by their LCC in their (continued) participation in sports betting. Also, they tend to overestimate how much others bet, and they approve of betting-related activities. Seal et al.’s study also found that sports bettors, relative to non-bettors or non-sports bettors, may have a false consensus about betting participation in general (i.e., biased social norms), as they think most people in society bet on sports [45]. Sports bettors believe that their family members and friends are supportive of their sports betting behaviors. Especially, sports bettors are surrounded by groups of friends who bet on sports and discuss sports betting regularly [12, 23, 47]. Accordingly, preventive efforts should focus on changing college students’ misperceptions concerning sports betting and how to effectively manage peer pressure for sports betting [48]. Strong social support (i.e., social norm) is found to be a strong protective factor against problem betting and pathological gambling [49, 50]. Thus, parents and people important to college students should play a critical role as protective agents for young adults with potential betting and gambling issues.

College students with a lower PBC on resisting sports betting are more likely to have a higher intention to bet on sports and engage in problem betting behaviors [12]. This study could not find a significant influence of PBC on intention, but as hypothesized, PBC was negatively related to problem betting among college students. Regarding PBC, one of the major issues is college students’ overestimation of their ability to control their betting participation and outcomes. Overestimating the chances of winning is one of the most critical predictors of problem betting. Accordingly, interventional efforts should be directed for bettors not to overestimate their chances of winning [51] and how to control their betting behavior. Intoxicated betting (e.g., betting when drunk or high) is especially harmful to those between the ages of 18–24, leading to increased risk-taking and spending and harmful personal relationships [9]. Thus, educational and health promotion agencies should educate college students to empower their perceived control over sports betting and educate them on the harmful consequences of intoxicated betting.

College students’ impulsive sports betting tendencies and previous betting experiences played a moderator role in the relationships between the intention and program betting. The intention-PSB relationship was stronger with the low impulsivity group and the high experience group. While the intention to bet is the most critical predictor of sports betting and potential program betting, other potential determinants, and moderators, such as habitual conduct, personality variables (e.g., sensation seeking), substance consumption, and impulsivity, should be concurrently considered in understanding college students’ problem betting [52]. Young adults have a tendency to chase their losses and bet more than they can afford, especially when they bet online [53]. More recently, young adults bet online and bet on e-sports, as e-sports betting services have proliferated in recent years [53]. As online betting, relative to traditional in-person wagering, is significantly more associated with impulsive betting and problem betting, future studies should examine the influence of sports betting categories (i.e., wagering on traditional sports vs. e-sports).

The theoretical framework of the current study was TPB, given its utility and applicability in understanding betting-related behavior. Even though the current study included extended variables, especially moderators, in understanding college students’ sports betting behavior, other potentially meaningful constructs and variables, such as personality traits or substance consumption, should be considered in future studies [22, 26].

Several limitations to this study should be noted for future studies. Firstly, the current study utilized a cross-sectional research design with a convenience sampling method. Also, each state in the U.S. has different legal guidelines concerning sports betting [15]. Therefore, the results of the current study might not be generalizable to other college populations in other countries. Secondly, while all the measures used in this study were reliable and valid, one of the measures used in this study showed a less-than-desirable reliability value (i.e., a Cronbach’s alpha of 0.65, instead of 0.70). Future studies should consider using more reliable scales from the literature. While there are several limitations, overall, the proposed model in this study could be used as a solid basis for future studies in sports betting, given that the current study used the TPB as a conceptual framework while incorporating non-volitional variables (e.g., impulsive betting tendency). Consequently, this study can contribute to developing better educational and interventional programs for sports betting and problem betting with a better understanding of college bettors.

### Electronic supplementary material

Below is the link to the electronic supplementary material.

Supplementary Material 1

## Data Availability

The datasets generated and analyzed in this study are not publicly available due to confidentiality and privacy-related issues but are available from the corresponding author on reasonable request.
