# Supplementary material for: Determinants of problem sports betting among college students: moderating roles of betting frequency and impulsive betting tendency
Source: BMC Psychol. 2023 Oct 23;11:352. doi: 10.1186/s40359-023-01387-w (PMC10594762; doi:10.1186/s40359-023-01387-w)
Supplement: Supplementary file 1 — Supplementary Material 1 [file 40359_2023_1387_MOESM1_ESM.docx]

Appendix A: Construct reliability and validity

| Construct / Item | Factor loading |
| --- | --- |
| Attitude (α = .827, CR = .878, AVE = .592) |  |
| ATT 1. Betting on sports can be an easy way to make money. | .754 |
| ATT 2. It is possible to consistently profit from sports betting. | .823 |
| ATT 3. Sports betting makes actual sports games more exciting. | .729 |
| ATT 4. Sports betting can be a good leisure activity. | .748 |
| ATT 5. I found betting on sports enjoyable. | .789 |
| Normative beliefs (α = .718, CR = .878, AVE = .706) |  |
| NB 1. People important to me think that it is okay to bet on sports. | .851 |
| NB 2. People important to me would approve of sports betting. | .871 |
| NB 3. Most people important to me bet on sports sometimes. | .795 |
| Motivation to comply (α = .716, CR = .876, AVE = .780) |  |
| MC 1. If people important to me bet on sports, I would also bet on sports. | .899 |
| MC 2. If friends/colleagues ask me to join a sports betting pool, I would also participate. | .867 |
| PBC (α = .653, CR = .804, AVE = .672) |  |
| PBC 1. Whether or not I bet on sports is entirely up to me. | .810 |
| PBC 2. I feel that I have complete control over betting on sports. | .829 |
| Sports betting intention (α = .888, CR = .947, AVE = .899) |  |
| INT 1. I intend to bet on sports near future. | .950 |
| INT 2. I intend to continue betting on sprots in the future. | .946 |
| Problem sports betting (α = .840, CR = .874, AVE = .636) |  |
| PSB 1. I have at times betted on sports more than I intended to. | .858 |
| PSB 2. I often try to win back on another day the money I lose in sports betting. | .826 |
| PSB 3. On occasions I have borrowed money for sports betting secretly from family or friends. | .692 |
| PSB 4. Sometimes I try to keep the amount I bet on sports secret from family or friends. | .804 |
| Impulsive sports betting (α = .773, CR = .826, AVE = .615) |  |
| ISBT 1. I am a person who makes unplanned bets on sports. | .875 |
| ISBT 2. It is fund to bet on sports spontaneously. | .659 |
| ISBT 3. I often bet on sports even if I had not intended to do. | .804 |

Note. α = Cronbach’s alpha, CR = Composite reliability, AVE = Average variance explained
